# SEOM-GEICO Clinical Guidelines on cervical cancer (2023)

**DOI:** 10.1007/s12094-024-03604-3

**Published:** 2024-08-31

**Authors:** Luis Manso, Avinash Ramchandani-Vaswani, Ignacio Romero, Luisa Sánchez-Lorenzo, María José Bermejo-Pérez, Purificación Estévez-García, Lorena Fariña-Madrid, Yolanda García García, Marta Gil-Martin, María Quindós

**Affiliations:** 1 Medical Oncology Department, Hospital Univ. 12 de Octubre, Madrid, Spain; 2 Medical Oncology Department, Hospital Universitario Insular de Gran Canaria, Gran Canaria, Spain; 3 Medical Oncology Department, Instituto Valenciano de Oncología, Valencia, Spain; 4 Medical Oncology Department, Clínica Universidad de Navarra, Madrid, Spain; 5 Medical Oncology Department, UGCI Oncol. Hosp Univer Regional y Virgen Victoria, IBIMA, Málaga, Spain; 6 Medical Oncology Department, Instituto de Biomedicina de Sevilla (IBIS), University Hospital Virgen del Rocío, Seville, Spain; 7 Medical Oncology Department, Vall d’Hebron Institute of Oncology, Vall d’Hebron University Hospital, Barcelona, Spain; 8 Medical Oncology Department, Institut d’Investigació i Innovació Parc Taulí (I3PT), arc Taulí Hospital Universitari, Universitat Autònoma de Barcelona, Sabadell, Spain; 9 Medical Oncology Department, Institut Català d’Oncologia i’Hospitalet, Hospitalet de Llobregat, Spain; 10 Medical Oncology Department, Complexo Hospitalario Universitario de A Coruña. Biomedical Research Institute (INIBIC), A Coruña, Spain

**Keywords:** Cervical cancer, Guideline, Diagnosis, Treatment

## Abstract

Cervical cancer (CC) is the fourth most common cancer and the fourth leading cause of mortality in women worldwide. It is strongly associated with high-risk human papillomavirus infection. High-income countries that have implemented human papillomavirus (HPV) vaccination and screening programs have seen dramatic reductions in CC incidence, while developing countries where these programs are not available continue to experience high rates of CC deaths. In early-stage CC, the primary treatment is surgery or radiotherapy, whereas concurrent chemo-radiotherapy (CRT) remains the conventional approach in locally advanced stages until the upcoming approval of immunotherapy. The incorporation of immunotherapy in combination with chemotherapy (with or without bevacizumab) in first line and as monotherapy in second line after platinum-based chemotherapy, has significantly increased overall survival (OS) in recurrent or metastatic CC. The purpose of this guideline is to summarize the most relevant evidence in the diagnosis, treatment, and follow-up of CC and to provide evidence-based recommendations for clinical practice.

## Incidence and epidemiology

Cervical cancer (CC) is the fourth most common cancer among women globally. In 2022, there were 661,021 new cases diagnosed worldwide: 61.072 in Europe and 1.679 in Spain [1].

Globally, a total of 350,000 deaths were reported in 2022. Approximately 90% of all new cases and deaths reported worldwide occurred in low- and middle-income countries. The 5-year relative survival for women diagnosed with CC in 2013 and 2019 was 67.2% [1].

The variation in CC rates across different geographic regions can be attributed to disparities in the prevalence of human papillomavirus (HPV) infection, a major risk factor for CC, as well as to differences in screening availability and limited access to vaccination in transitioning countries [2].

HPV, and the oncogenic subtypes HPV16 and 18 in particular, is detected in around 99% of cervical tumors. Prophylactic administration of the HPV vaccine to females aged 9 through 12 has proven to be an effective measure in preventing HPV infection and related diseases. As a result, several countries have implemented HPV vaccination programs [3, 4] **[II, A]**.

On the other hand, advances in secondary prevention, with the introduction of highly sensitive HPV DNA testing, has improved the effectiveness of traditional Papanicolaou cytology in screening programs. This development has improved secondary prevention methods intended to diagnose CC at an early stage and prevent its progression [5, 6] **[II, A]**.

## Methodology

This guideline is based on a systematic review of relevant published studies with the consensus of ten treatment expert oncologists from GEICO (the Spanish Gynaecological Cancer Research Group), SEOM (the Spanish Society of Medical Oncology), and an external review panel of two experts designated by SEOM. The Infectious Diseases Society of America-US Public Health Service Grading System for Ranking Recommendations in Clinical Guidelines has been used to assign levels of evidence and grades of recommendation (Table 1) [7].

**Table 1:** Levels of evidence/ grades of recommendation

Levels of evidence
I. Evidence from at least one large randomized, controlled trial of good methodological quality (low potential for bias) or meta-analyses of well-conducted randomized trials without heterogeneity
II. Small randomized trials or large randomized trials with a suspicion of bias (lower methodological quality) or meta-analyses of such trials or of trials with demonstrated heterogeneity
III. Prospective cohort studies
IV. Retrospective cohort studies or case–control studies
V. Studies without a control group, case reports, and expert opinions

Grades of recommendation
A. Strong evidence for efficacy with a substantial clinical benefit; strongly recommended
B. Strong or moderate evidence for efficacy, but with a limited clinical benefit; generally recommended
C. Insufficient evidence for efficacy or benefit that does not outweigh the risk or the disadvantages; optional
D. Moderate evidence against efficacy or for adverse outcome; generally not recommended
E. Strong evidence against efficacy or for adverse outcome; never recommended

## Diagnosis

Early CC is frequently asymptomatic, underscoring the importance of screening. Abnormal cervical cytology or a positive high-risk HPV test should prompt the performance of colposcopy and biopsy, or excisional procedures such as loop electrosurgical excision and conization.

Sometimes, incidentally visible lesions are discovered upon pelvic examination. Carcinomas can be exophytic, growing out of the surface, or endophytic with stromal infiltration and minimal surface growth. If a gross palpable lesion is present, diagnosis is based on biopsy. If a thorough pelvic examination cannot be carried out or there is uncertainty regarding vaginal/parametrial involvement, it should preferably be conducted under anesthesia [7].

Locally advanced CC (LACC) may cause abnormal vaginal bleeding or discharge, pelvic pain, and dyspareunia. These symptoms are non-specific and may be mistaken for vaginitis or cervicitis.

Some patients present with pelvic or lower back pain, which may radiate along the posterior side of the lower extremities. Bowel or urinary symptoms, such as pressure-related complaints, hematuria, hematochezia, or the passage of urine or stool through the vagina, are uncommon and point toward advanced disease [8].

## Pathology and molecular biology

The World Health Organization (WHO) recognizes three categories of epithelial tumors of the cervix: squamous, glandular and other epithelial tumors, along with mixed epithelial, mesenchymal tumors and germ cell tumors [9]. Squamous cell carcinoma (SCC) accounts for approximately 80% of all CC, while adenocarcinoma (ADC) accounts for some 20% [9].

Historically, the development of all CC has been regarded as being associated with HPV infection (HPV-A). Nevertheless, it has recently been recognized that a significant proportion of cervical ADC are HPV-independent (HPV-I) [10]. HPV status is both a prognostic and predictive factor. HPV-A tumors entail better prognosis and better response to treatment compared with HPV-I tumors [10]. Therefore, the latest WHO classification of lower genital tract tumors in 2020 categorizes CC into HPV-A and HPV-I [11].

Hight-risk HPV genotypes cause the vast majority (> 95%) of SCC. Twelve HPV types are classified by WHO as oncogenic: 16, 18, 31, 33, 35, 39, 45, 51, 52, 56, 58, and 59. However, two types (16 and 18) alone are responsible for 70% of all SCC.

The HPV viral oncoproteins E6 and E7 inactivate p53 and RB1, respectively. This inactivation is associated with the integration of HPV into the host genome, resulting in genomic instability and the accumulation of somatic mutations. Several factors have been linked to an increased risk of HPV persistence and progression, including immunosuppression (particularly due to human immunodeficiency virus), multiparity, smoking, and the use of oral contraceptives [9].

### Squamous cell carcinoma

These tumors arise in dividing epithelial cells in high-grade squamous intraepithelial lesions (HSIL), a so-called transforming infection. The progression of high-grade lesions to SCC requires the accumulation of additional, yet incompletely understood, genetic and epigenetic alterations, a process that may take up to 20–30 years.

While HPV analysis is not necessary for diagnosis, p16 immunoreactivity can serve as a surrogate marker for high-risk HPV infection [12].

More than 70% of HPV-A SCC exhibit genomic alterations in either one or both of the PI3K/MAPK and TGF-β signaling pathways. Genes such as ERBB3 (HER3), CASP8, HLA-A, SHKBP1, and TGFBR2 have been reported as significantly mutated [13].

### Adenocarcinoma

ADC encompasses a heterogeneous group of tumors. Most are HPV-A (typically types 18, 16, and 45), although around 10–15% are HPV-I [10]. The usual type accounts for about 75% of all ADCs, while the mucinous type represents some 10%. HPV-A ADCs tend to have low levels of copy-number alterations and low scores for epithelial-mesenchymal transition. KRAS mutations are common [9]. All forms of invasive HPV-A ADCs can exhibit either destructive or non-destructive (ADC in situ) growth patterns. This classification has revealed associations between tumor invasive patterns and risk of nodal metastases, recurrence, and survival [14].

### Other histologies

Rare cervical cancer histologies include adenosquamous carcinoma, neuroendocrine tumors (small cell and large cell neuroendocrine carcinoma), rhabdomyosarcoma, primary cervical lymphoma, and cervical sarcoma. Accurate histological identification using specific markers is essential for optimal patient management.

### Predictive biomarkers in cervical cancer

Programmed Death-Ligand 1 (PD-L1) expression is a biomarker that predicts benefit from immune checkpoint inhibitors in patients with cervical cancer. Additionally, PD-L1 expression is more prevalent in squamous cell carcinomas compared to adenocarcinomas. Recommendations for patients with recurrent, progressive, or metastatic disease [13]:

- *PD-L1 Testing*: PD-L1 expression testing is recommended in patients with recurrent, progressive, or metastatic cervical cancer.
- HER-2 Immunohistochemistry (IHC) Testing: should be conducted to identify patients who may benefit from HER-2 targeted therapies.
- *Mismatch Repair (MMR) Testing*: MMR status can be evaluated using IHC.
- *Next-Generation Sequencing (NGS)*: may be contemplated to assess microsatellite instability (MSI) and tumor mutational burden (TMB), which can provide additional insights into potential therapeutic options.

## Staging and risk assessment

Since the beginning of the FIGO (The International Federation of Gynecology and Obstetrics) staging system, physical examination has been the primary tool for staging purposes. However, the latest FIGO update in 2018 incorporates imaging and pathology findings to improve the prognostic correlation and better tailor treatment [11] (Table 2).

**Table 2:** A 2018 FIGO staging

Stage description
I The carcinoma is strictly confined to the cervix (extension to the corpus should be disregarded). IA Invasive carcinoma that can be diagnosed only by microscopy with maximum depth of invasion ≤5 mm^a^ IA1 Measured stromal invasion ≤3 mm in depth IA2 Measured stromal invasion >3 mm and ≤5 mm in depth IB Invasive carcinoma with measured deepest invasion >5 mm (greater than stage IA); lesion limited to the cervix uteri with size measured bymaximum tumor diameter^b^ IB1 Invasive carcinoma >5 mm depth of stromal invasion and ≤2 cm in greatest dimension IB2 Invasive carcinoma >2 cm and ≤4 cm in greatest dimension IB3 Invasive carcinoma >4 cm in greatest dimension
II The cervical carcinoma invades beyond the uterus, but has not extended onto the lower third of the vagina or to the pelvic wall IIA Involvement limited to the upper two-thirds of the vagina without parametrial invasion IIA1 IIA1 Invasive carcinoma ≤4 cm in greatest dimension IIA2 Invasive carcinoma >4 cm in greatest dimension IIB With parametrial invasion but not up to the pelvic wall
III The carcinoma involves the lower third of the vagina and/or extends to the pelvic wall and/or causes hydronephrosis or non-functioning kidney and/or involves pelvic and/or paraaortic lymph nodes IIIA Carcinoma involves lower third of the vagina, with no extension to the pelvic wall IIIB Extension to the pelvic wall and/or hydronephrosis or non-functioning kidney (unless known to be due to another cause) IIIC Involvement of pelvic and/or paraaortic lymph nodes (including micrometastases),^c^ irrespective of tumor size and extent (with r and p notations). IIIC1 Pelvic lymph node metastasis only IIIC2 Paraaortic lymph node metastasis
IV The carcinoma has extended beyond the true pelvis or has involved (biopsy proven) the mucosa of the bladder or rectum. A bullous edema, as such, does not permit a case to be allotted to stage IV IVA Spread of the growth to adjacent organs IVB Spread to distant organs

^a^Imaging and pathology can be used, when available, to supplement clinical findings with respect to tumor size and extent, in all stages. Pathological findings supersede imaging and clinical findings

^b^The involvement of vascular/lymphatic spaces should not change the staging. The lateral extent of the lesion is no longer considered

^c^Isolated tumor cells do not change the stage but their presence should be recorded

Recommended radiological imaging include pelvic magnetic resonance imaging (MRI) to evaluate local disease extension (preferred for FIGO stage IB1–IB3). Additionally, positron emission tomography/computed tomography (PET/CT) in early stages with suspicious lymph nodes (LN) or locally advanced tumors (EIB3 and higher) is recommended to assess nodal and distant disease. If PET/CT is not available, chest and abdominal CT can be used instead **[II, B]**.

Cystoscopy and proctoscopy are only recommended if bladder or rectal invasion is suspected **[IV, D]**.

Sentinel lymph node (SLN) mapping is especially relevant for staging early-stage cervical cancer (FIGO stages IA1 with lymphovascular space invasion, IA2, and IB1). SLNs should undergo ultrastaging to detect low-volume metastasis; non-sentinel nodes do not require ultrastaging.

Para-aortic lymph nodes (PALN) evaluation has been the object of debate in recent years. PALN involvement is closely related to pelvic LN metastasis and tumors > 2 cm. Surgical staging versus PET/CT for patients with no suspicious radiological pelvic LN invasion has been evaluated, given that it can modify the extension of the radiotherapy field. Most evidence is retrospective [15], and some randomized trials were prematurely closed or patients with suspicious LN were included [16]. These studies showed that surgery identified more PALN metastases, but without a clear benefit in OS compared with PET/CT staging. A randomized trial has been recently initiated, designed to demonstrate whether para-aortic lymphadenectomy followed by tailored chemoradiation improves results compared to patients staged with FDG-PET/CT only followed by chemoradiation [17].

Therefore, PALN dissection may be considered to reduce the risk of undetected occult metastases when imaging shows no PALN involvement **[II, B]**.

Tumor risk assessment includes several factors including tumor size, stage, depth of tumor invasion, LN status, lymphovascular space invasion (LVSI), and histological subtype [18]. These factors have been included in trials to indiviualize the best adjuvant treatment. The “Sedlis Criteria” (GOG-092 trial) identify intermediate-risk factors: deep stromal invasion (> 1/3), lymphovascular space involvement, or tumor size > 4 cm [19]. The GOG-109 trial identified high-risk factors: positive LN, positive margins, and/or microscopic parametrial involvement [20].

According to the SEER database 2022, the 5-year survival rates are 91% for early stages, 60% for locally advanced stages, and 19% for metastatic cases. Kristensen et al. reported that the 5-year survival rate was better for patients with smaller tumors (94.8% if < 2 cm and 79.1% if 2–3.9 cm) [21]. Five-year survival is < 50% in patients with pelvic LN metastasis and < 20–30% in those with PALN metastasis.

## Management of local and locoregional disease

### Early-stage disease

- T1a1 disease: conization with negative margins should be considered [IV, C]. Sentinel lymph node (SLN) biopsy is worth considering in LVSI-positive cases **[IV, B]**.
- T1a2 disease: conization with clear margins or a simple hysterectomy (HT) is deemed sufficient **[IV, B]**. While SLN biopsy can be contemplated for LVSI-negative patients, it is recommended for those with LVSI-positive cases **[IV, B]**.

### Management of T1b1, T1b2, and T2a1 disease

- For patients diagnosed with stage IB1, IB2, or IIA1, surgery stands as the most suitable choice **[I, A]**. The initial surgical step should involve LN staging **[IV, A]**. SLN mapping and any suspicious nodes should be removed intraoperatively **[III, A]**.
- If any LN involvement is detected intraoperatively, refrain from further surgical procedures, opting instead for definitive concurrent chemoradiotherapy (CRT) **[III, A]**. In these cases, consider para-aortic lymph node dissection (PALND) for staging purposes **[IV, C]**.
- If both sides reveal negative SLN in pelvic level I, LN dissection can be confined to level I **[IV, B]**.
- When SLN is not detected on either side, LN dissection should include the usual areas: obturator fossa, external iliac regions, common iliac regions, and presacral region **[III, A]**.

Based on the LACC trial findings, laparotomy remains the recommended approach for radical parametrectomy procedures due to the higher risk of relapse associated with minimally invasive surgery (MIS) **[I, A]** [22]. However, a retrospective multicenter study found no increased risk of relapse associated with minimally invasive surgery (MIS) in a low-risk group of patients with small tumors (< 2 cm) following conization with clear margins and with MIS being regarded as acceptable **[IV, C]** [23]. The recent SHAPE study suggests that for early-stage, low-risk cervical carcinoma (FIGO stages [2018] IA2–IB1 ≤ 2 cm with limited stromal invasion), simple total HT could be considered, inasmuch as it has demonstrated non-inferiority to radical HT in 3-year pelvic recurrence, recurrence-free survival, or overall survival (OS) rates [24].

When surgery is not feasible, consider definitive CRT and brachytherapy (BT) **[IV, B]**.

### Fertility-sparing treatment

Fertility-sparing therapy is suitable for young patients with tumors < 2 cm (stage IA and IB1), with squamous cell carcinoma or HPV-related ADC **[III, B]**. A thorough counseling on disease and pregnancy risks is recommended. Approaches vary depending on tumor stage and LVSI status. In T1a1/T1a2/T1b1 tumors both conization and simple trachelectomy can be recommended, regardless of LVSI presence **[IV, B]**, while in T1b1, radical trachelectomy remains an option **[IV, B]** but is strongly advised in LVSI-positive cases **[III, B]**. LN staging is recommended following the principles of early-stage management. **[III, B]**.

## Postoperative adjuvant treatment

### Intermediate risk

In the absence of positive LN, pathology risk factors in the surgical specimens include size > 4 cm, deep cervical stromal invasion, and positive LVSI. According to Sedlis criteria, when two or more of these features are identified, CC is classified as intermediate risk. This group of patients are treated disparately around the world, including study limitations that do not include other significant risk factors, such as histology and proximal margins, currently considered in the present landscape of CC treatment.

In the original GOG-092 trial, 277 patients with two or more risk features were randomized to observation vs external beam radiation therapy (EBRT). With a median follow-up of 10 years, a significant benefit was demonstrated in terms of progression free-survival (PFS) HR 0.54, 95% CI 0.35–0.81, *p* = 0.007, albeit not OS (HR 0.7; *p* = 0.07) **[II, B]** [19]. The role of chemotherapy (ChT) in this population is presently the object of research in the GOG-263 trial.

### High risk

If positive pelvic LN, positive surgical margins, and/or positive parametrium are identified, postoperative pelvic EBRT with concurrent platinum-containing ChT is recommended. In the GOG-109 trial, 268 women with IA2, IB, and IIA stage CC received adjuvant radiotherapy (RT) with or without ChT (cisplatin–5-fluorouracil) for 4 courses. The study evidenced that the ChT arm achieved better 4-year OS (81% vs. 71%) and PFS (80% vs. 63%) outcomes **[I, A]** [20]. That being said, the current ChT regimen of choice is weekly cisplatin. The cisplatin dosage for this schedule is 40 mg/m^2^ per week, with a 70-mg weekly limit based on other concurrent trials in locally advanced disease.

## Locally advanced disease

Locally advanced disease is defined as FIGO stages IB2, II, III, and IVA. Radical treatment with EBRT and weekly cisplatin followed by brachytherapy has demonstrated benefit in five, phase 3 trials, as well as in a Cochrane meta-analysis [25]. This approach results in a 10% increase in OS and a 50% decrease in the risk of relapse and is the current standard of care **[I, A]**. The alternative in case of renal impairment could be weekly carboplatin AUC2.

### Adjuvant chemotherapy

The potential role of adjuvant ChT following a concurrent treatment modality has been addressed in two, phase 3 trials with contradictory results and has not been endorsed as standard treatment. While adding two cycles of adjuvant carboplatin and gemcitabine increased PFS and OS, toxicity was an issue. On the other hand, the OUTBACK trial, with four cycles of carboplatin and paclitaxel, failed to increase either PFS or OS [26]. The main concerns have consistently been the low adherence of ChT after concurrent treatment and toxicity.

### Addition of immunotherapy

The KEYNOTE-A18 study has recently been published that examined the efficacy of adding pembrolizumab to standard CRT in patients with high-risk, locally advanced CC (FIGO 2014 stage IB2-IIB with node-positive disease or stage III-IVA). The results revealed an increase in PFS with a HR 0.70 (0.55–0.89, *p* = 0.0020) and 24-month OS of 87% in the pembrolizumab-chemoradiotherapy group and 81% in the placebo-chemoradiotherapy group. On 12 January 2024, the Food and Drug Administration (FDA) approved pembrolizumab with chemoradiotherapy (CRT) for patients with FIGO 2014 stage III-IVA CC and is now under review by the European Medicines Agency [27]. Therefore, the addition of pembrolizumab to CRT will likely become the standard treatment for LACC in the near future **[I, A]**. *This combination is not approved by the European Medicines Agency (EMA) for cervical cancer and is not reimbursed by the Spanish public healthcare system, at the time of writing this document.*

### Neoadjuvant/induction chemotherapy

The Neoadjuvant/Induction ChT approach has been addressed in two different settings:

The first one is to make locally advanced CC amenable to surgery and compares ChT followed by surgery to standard concurrent CRT. Two large, phase 3 trials failed to prove improved OS and a metanalysis that included smaller studies has not modified standard treatment [28, 29].

The second approach involves induction ChT in LACC prior to standard CRT compared to CRT. The GCIG INTERLACE trial randomized 500 IB1 node positive, IB2, II, IIIB, and IVA (FIGO 2008) patients to 6 cycles of weekly carboplatin (AUC 2) and paclitaxel (80 mg/m^2^) before CRT versus CRT alone. This has shown a 5-year PFS rate of 73% and OS rate of with induction ChT prior to CRT compared to 64% (HR 0.65; 95% CI 0.46–0.91; *p* = 0.013) and 72% (HR 0.61; 95% CI 0.40–0.91; *p* = 0.04), respectively, with CRT alone **[I, B]** [30].

#### Recommendations

- Primary weekly cisplatin-based (40 mg/m^2^) CRT remains the standard of care for LACC until immunotherapy is approved **[I, A]**.
- Induction chemotherapy with the INTERLACE regimen before definitive CRT might be an option for selected patients **[I, B]**.
- Adjuvant ChT after CRT is not recommended **[I, D]**.
- Neoadjuvant ChT before radical surgery is not a standard approach in LACC **[I, D]**.
- The addition of pembrolizumab to CRT will likely become the standard treatment for LACC **[I, A]**.

## Locoregional recurrent disease

Patients suspected of recurrent disease require a thorough diagnostic work-up and the recurrence should be histologically confirmed.

### Central pelvic recurrence

The recommended treatment following primary surgery includes definitive CRT combined with BT. External boost techniques should not replace BT. For previously irradiated patients, pelvic exenteration based on tumor location is suggested. This recommendation is typically reserved for referral centers with specialized expertise in managing persistent or recurrent CC cases. Reirradiation should be selectively weighed, considering factors such as disease volume, time since prior RT, and total dose administered.

### Pelvic sidewall recurrence

After primary surgery, CRT is the preferred option. If not feasible, extensive pelvic surgery should be considered, including intra-operative RT or when free surgical margins are not feasible. For those who received prior RT, extensive pelvic surgery is the first option. Patients ineligible for surgery due to comorbidities or a low probability of complete resection should receive systemic ChT.

#### Recommendations

- Pelvic exenteration is recommended for central pelvic recurrence where there is no involvement of the pelvic sidewall, extrapelvic nodes, or peritoneal disease **[IV, B].**
- Reirradiation for central recurrences could be considered in selected cases. This must be performed only in specialized centers **[IV, C].**
- In patients with pelvic sidewall involvement, extended pelvic surgery can be considered in specialized centers **[IV, B].**
- Patients who are not candidates for extensive surgery should be treated with systemic chemotherapy **[IV, B].**

## Management of advanced and metastatic disease

The risk of recurrence ranges from 16 to 30% in early stages and up to 70% in LACC. Most relapses occur within the first two years after diagnosis and 50–60% of patients will have disease beyond the pelvis. Subjects who develop distant metastases, either at initial presentation or at relapse, are rarely curable. For highly selected patients with isolated distant metastases amenable to local treatment, occasional long-term survival has been reported [31].

ChT is often recommended for patients with extrapelvic metastases or recurrent disease who are not candidates for RT or exenterative surgery.

### First-line treatment

Cisplatin has been regarded as the most effective agent for metastatic CC [32]. Cisplatin-based doublets with topotecan or paclitaxel have demonstrated superiority over cisplatin monotherapy in terms of response rate and PFS [33, 34]. Cisplatin/paclitaxel is less toxic than cisplatin/topotecan and is considered the regimen of choice **[II, B]** [35].

Tumor angiogenesis plays a significant role in CC. The GOG240 phase III trial examined the addition of bevacizumab to combination ChT regimens in the first line metastatic setting (cisplatin/paclitaxel or topotecan/paclitaxel) in 452 patients with metastatic, persistent, or recurrent CC in the context of first-line treatment [36]. The study revealed significant improvements in OS among patients receiving bevacizumab (16.8 months vs 13.3 months; HR 0.77; 95% CI 0.62–0.95;* p* = 0.007). Additionally, data from a phase III randomized trial (JCOG0505) suggested that carboplatin/paclitaxel was non-inferior to cisplatin/paclitaxel in 253 patients with metastatic or recurrent CC [37]. However, cisplatin remains the key drug for patients who have not previously received platinum agents. Furthermore, the phase II CECILIA trial proved that bevacizumab can be safely combined with carboplatin-paclitaxel, with the incidence of fistula/gastrointestinal perforation aligning with that observed in the GOG240 study [38].

Given these results and based on the balance between efficacy and toxicity, paclitaxel and platinum ChT combined with bevacizumab was deemed the regimen of choice in first-line metastatic or recurrent CC.

Programmed death ligand 1 (PD-L1) also plays a role in CC pathogenesis [39]. In the phase II KEYNOTE-158 trial, an objective response to pembrolizumab was noted in 14.3% of patients with PD-L1 positive tumors who had received > 1 prior ChT regimens for recurrent or metastatic disease [40]. *This treatment is not approved by the European Medicines Agency (EMA) for cervical cancer and is not reimbursed by the Spanish public healthcare system, at the time of writing this document.*

The results of the KEYNOTE-826 trial displayed that PFS and OS were significantly greater with pembrolizumab than with placebo among patients with persistent, recurrent, or metastatic CC who were also receiving platinum-based chemotherapy with or without bevacizumab. The addition of pembrolizumab significantly improved PFS (10.4 months vs 8.2 months HR 0.62; 95% CI 0.50–0.77, *p* < 0.001) and OS (28.6 vs 16.5 months HR 0.60; 95% CI 0.49–0.74), leading to regulatory approval of pembrolizumab for persistent, recurrent, or metastatic CC tumors expressing PD-L1 with a combined positive score (CPS) ≥ 1 **[I, A]**. In the small subgroup of patients with a CPS < 1, the hazard ratios of PFS and OS were close to 1. Given the small size of that subgroup (11.2% of the patients), the effect of adding pembrolizumab appears to be small [41, 42].

Recently, in the phase III BEATcc trial, patients with metastatic (stage IVB), persistent, or recurrent CC were randomly assigned in a 1:1 ratio to receive bevacizumab plus platinum and paclitaxel, with or without atezolizumab. BEATcc evaluated the PD-L1 inhibitor atezolizumab in a biomarker-unselected population and the use of bevacizumab was mandatory. Median PFS was 13.7 months with atezolizumab compared to 10.4 months with standard therapy (HR 0.62; 95% CI 0.49–0.78, *p* < 0.0001). Median OS was 32.1 months with atezolizumab compared to 22.8 months with standard therapy (HR 0.68; 95% CI 0.52–0.88, *p* = 0.0046) [43]. *This combination is not approved by the European Medicines Agency (EMA) for cervical cancer and is not reimbursed by the Spanish public healthcare system, at the time of writing this document.*

### Second-line and single agents

In patients progressing to first-line therapy, several chemotherapies, such as vinorelbine, topotecan, gemcitabine, or paclitaxel, have been examined. However, response rates to these treatments were very low (10–13%) and had a short duration.

To determine if the immune checkpoint inhibitors after failure to platinum therapy were superior to standard ChT in terms of OS, the phase 3 GOG 3016/ENGOT-cx9 (EMPOWER Cervical-1) trial randomized 608 patients to receive cemiplimab or investigator’s choice of intravenous ChT. Cemiplimab exhibited a statistically significant improvement in OS compared to ChT (12.0 months vs 8.5 months; HR 0.69; 95% CI 0.56–0.84; *p* < 0.001), in both SCC and the entire population, regardless of PD-L1 status. These results led to cemiplimab receiving regulatory approval as monotherapy to treat patients with recurrent or metastatic CC and disease progression on or after platinum-based ChT **[I, A]** [44].

Tisotumab vedotin (TV) is an antibody–drug conjugate that targets tissue factor. TV revealed promising and durable responses in the treatment of patients with recurrent or metastatic CC in a phase 2 study, which led to its accelerated approval in the US [45]. Recently, the global phase III innovaTV 301/ENGOT-cx12/GOG-3057 trial, randomized patients with recurrent or metastatic CC with progression on or after standard of care to TV monotherapy or the investigator’s choice of chemotherapy. The TV arm exhibited a 30% reduction in risk of death vs chemotherapy (HR 0.70; 95% CI 0.54–0.89; *p* = 0.0038), with significantly longer median OS (11.5 months vs 9.5 months). *This treatment is not approved by the European Medicines Agency (EMA) for cervical cancer and is not reimbursed by the Spanish public healthcare system, at the time of writing this document.*

#### Recommendations

**Fig. 1:**
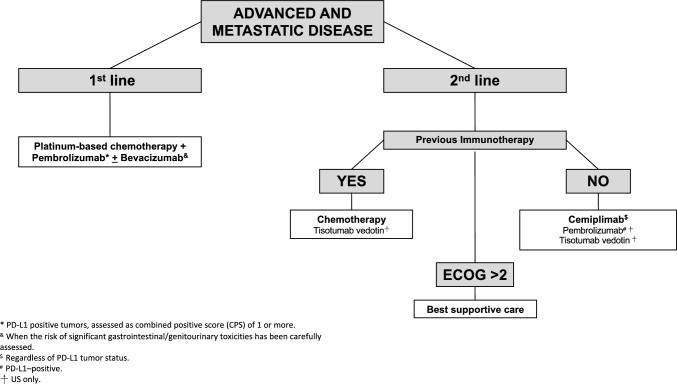
Recurrent and metastatic disease

- Platinum-based ChT combined with pembrolizumab is recommended for medically fit patients with recurrent/metastatic PD-L1 positive CC, assessed as CPS of 1 or more **[I, A].**
- Carboplatin/paclitaxel and cisplatin/paclitaxel are the preferred regimens **[I, A].**
- The addition of bevacizumab is recommended when the risk of significant gastrointestinal/genitourinary fistula has been carefully assessed and discussed with the patient **[I, A].**
- Patients who progress after first-line platinum-based ChT and have not yet received immunotherapy should be offered cemiplimab, regardless of PD-L1 tumor status **[I, A]** (Fig. 1).

## Follow up, long-term implications, and survivorship

Follow-up recommendations in CC are based on the individual risk of recurrence depending on prognostic factors, treatment approach, and patient characteristics, although there is no current evidence of the most appropriate strategy.

Follow-up should be more thorough during the first 2–3 years after primary treatment, as this is when the majority of recurrences typically occur, especially in high-risk patients [46]. History and complete physical examination, including vaginal and pelvic-rectal examination performed by a specialist, are recommended at each visit. Systematic cervical and/or vaginal cytology after CRT or surgery has a low positive predictive value for detecting recurrence. HPV testing could be useful instead, albeit strong evidence is still lacking.

For high-risk patients with stage II or greater, CT or PET/CT (preferred) and pelvic MRI (recommended), should be performed within 3–6 months of completing therapy.

A reasonable follow-up schedule involves visits every 3–6 months during the first two years and every 6–12 months during years 3–5. Table 3 summarizes our recommendations for follow-up. The role of additional imaging has not been well established and should be guided by symptoms and clinical concern for suspected recurrent/metastatic disease. Patients should return to annual population-based general physical and pelvic examinations after five years of recurrence-free follow-up [47].

**Table 3:** Cervical cancer follow-up recommendations

	0–2 years from end of treatment	2–5 years from end of treatment	> 5 years from end of treatment
Medical history and general physical examination	Every 3–6 months*	Every 6 months	Yearly
Gynecological examination (including HPV)	Every 3–6 months*	Every 6 months	Yearly
Imaging (CT or PET-CT scan and pelvic MRI)**	Every 6 months	Every 6–12 months	Yearly

*Every 3–4 months in high-risk patients

**Recommended in high-risk patients or if clinically indicated

Following treatment, patients should be educated about signs/symptoms suggestive of recurrence as a relevant part of the surveillance plan.

Early use of vaginal dilators concurrent with lubricants and topical estrogen is recommended for suitable sexual rehabilitation. Patients should be informed about the possible benefits of healthy lifestyle habits in reducing the risk of recurrence and improving overall well-being.

### Additional table summary of recommendations

**Table:** 

Incidence and epidemiology
Prophylactic administration of the HPV vaccine has proven to be an effective measure in preventing HPV infection and related diseases **[II, A]** Introduction of highly sensitive HPV DNA testing has improved the effectiveness of traditional Papanicolaou cytology in screening programs **[II, A]**
Staging and risk assessment
Tumor risk assessment includes tumor size, stage, depth of tumor invasion, lymph node status, LVSI, and histological subtype. Lymph node status and number of lymph nodes involved are the most important prognostic factors
Management of local/locoregional disease
T1a1 disease: conization with negative margins could be considered [IV, C]. Sentinel lymph node (SLN) biopsy is worth considering in LVSI-positive cases **[IV, B]** T1a2 disease: conization with clear margins or a simple hysterectomy (HT) is regarded as sufficient treatment **[IV, B]**. While SLN biopsy can be contemplated for LVSI-negative patients, it is recommended for LVSI-positive cases **[IV, B]** For patients diagnosed with stage IB1, IB2, or IIA1, surgery stands as the most suitable choice **[I, A]**. The initial surgical step should involve LN staging **[IV, A]**. SLN mapping and any suspicious nodes should be removed intraoperatively **[III, A]** If any LN involvement is detected intraoperatively, refrain from further surgical procedures, opting instead for definitive concurrent chemoradiotherapy (CRT) **[III, A]**. In these cases, consider para-aortic lymph node dissection (PALND) for staging purposes **[IV, C]** If both sides show negative SLN in pelvic level I, LN dissection can be confined to level I **[IV, B]** When SLN is not detected on either side, LN dissection should cover traditional areas: obturator fossa, external iliac regions, common iliac regions, and presacral region **[III, A]** Laparotomy remains the recommended approach for radical parametrectomy procedures due to the higher risk of relapse associated with minimally invasive surgery (MIS) **[I, A]** Fertility-sparing therapy is suitable for young patients with tumors < 2 cm (stages IA and IB1), with squamous cell carcinoma, or HPV-related ADC **[III, B]** Primary weekly cisplatin-based (40 mg/m2) CRT remains the standard treatment in locally advanced CC until the approval of immunotherapy **[I, A]** Induction chemotherapy, following the INTERLACE regimen, before definitive CRT could be an option for selected patients **[I, B]** Adjuvant ChT after CRT is not recommended **[I, D]** Neoadjuvant ChT prior to radical surgery is not a standard approach in locally advanced CC **[I, D]** The addition of pembrolizumab to CRT will likely become the standard treatment for LACC **[I, A]**
Management of advanced/metastatic disease
Platinum-based ChT combined with pembrolizumab is recommended for medically fit patients with recurrent/metastatic PD-L1 positive CC, assessed as a CPS of ≥ 1 **[I, A]** Carboplatin/paclitaxel and cisplatin/paclitaxel are the preferred regimens **[I, A]** The addition of bevacizumab is recommended when the risk of significant gastrointestinal/genitourinary fistula has been carefully assessed and discussed with the patient **[I, A]** Patients who progress after first-line platinum-based ChT and have not yet received immunotherapy should be offered cemiplimab, regardless of PD-L1 tumor status **[I, A]**
Follow-up, long-term implications and survivorship
History and complete physical examination, including vaginal and pelvic-rectal examination performed by a specialist, are recommended at each visit A reasonable follow-up schedule involves visits every 3–6 months in the first two years and every 6–12 months in years 3–5. CT or PET/CT scan should be carried out as clinically indicated Patients should return to annual population-based general physical and pelvic examinations after five years of recurrence-free follow-up **[III, C]**

## Data Availability

Not applicable.
